# Epidemiological and clinical characteristics of severe acute respiratory coronavirus virus 2 (SARS-CoV-2) infection among healthcare workers in Hubei Province, China

**DOI:** 10.1017/ice.2020.1321

**Published:** 2020-11-18

**Authors:** Mingyang Wu, Cong Xie, Ran Wu, Yanling Shu, Lulin Wang, Mingyan Li, Youjie Wang

**Affiliations:** 1 Department of Maternal and Child Health, School of Public Health, Tongji Medical College, Huazhong University of Science and Technology, Wuhan, Hubei, China; 2 Institute of Preventive Medicine Information, Hubei Provincial Center for Disease Control and Prevention, Wuhan, Hubei, China; 3 Department of Laboratory Medicine, Wuhan Children’s Hospital, Tongji Medical College, Huazhong University of Science & Technology, Wuhan, Hubei, China

## Abstract

**Objective:**

To evaluate the epidemiological and clinical characteristics of severe acute respiratory coronavirus virus 2 (SARS-CoV-2) infection among healthcare workers (HCWs) in Hubei Province, China.

**Design:**

Retrospective cohort study.

**Setting:**

Hubei Provincial Center for Disease Control and Prevention.

**Participants:**

The participants in this study are cases identified by epidemiological investigation in Hubei Province, as of February 27, 2020, and were followed until March 7, 2020. In total, 1,989 HCWs and 41,137 other occupational cases were included for analysis.

**Methods:**

We used descriptive statistics to summarize patient characteristics.

**Results:**

Of 1,989 laboratory-confirmed HCWs, 297 (14.93%) had severe or critical cases, 73 (3.67%) had asymptomatic infections, and 18 died of coronavirus disease 2019 (COVID-19). The case fatality rate was 0.9%. The proportion of severe or critical cases decreased from the beginning to the end of the outbreak (from 21.29% to 3.52%), and the proportion of asymptomatic cases increased from 0.0% to 47.18%. Nearly half of HCWs with confirmed COVID-19 reported no known contact with COVID-19 patients (969, 48.72%). Fever and cough were the most common symptoms at disease onset in both HCWs and other occupational cases; however, HCWs had higher rates of fatigue (30.90% vs 25.02%; *P* < .001) and myalgia (19.15% vs 13.43%; *P* < .001). Additionally, compared with other occupational groups, HCWs were associated with a lower risk of death after adjustment for potential confounders (odd ratio [OR], 0.50; 95% confidence interval [CI], 0.30–0.79).

**Conclusions:**

Compared with COVID-19 cases in other occupational groups, HCWs with COVID-19 have half the risk of death, although they have been shown to have higher rates of fatigue and myalgia.

In December 2019, a series of unexplained pneumonia cases emerged in Wuhan, Hubei Province of China.^1^ The cause was later identified as a novel RNA β-coronavirus by deep sequencing analysis,^2^ and it has been named severe acute respiratory syndrome coronavirus 2 (SARS-CoV-2) by the International Committee on Taxonomy of Viruses.^3^ On February 11, 2020, the World Health Organization (WHO) officially named the disease caused by SARS-CoV-2 coronavirus disease 2019 (COVID-19).

COVID-19 has become a worldwide public health challenge. As of September 28, 2020, SARS-CoV-2 has affected 235 countries, causing >30 million infections and 991,224 deaths.^4^ Given the immediacy and widespread nature of the COVID-19 pandemic, healthcare workers (HCWs) around the world are facing increasing challenges and higher infection risks. A recent meta-analysis showed that among all COVID-19 patients in different countries, HCWs accounted for 4.2%–17.8% of cases.^5^ As of February 11, 2020, a report from the Chinese Centers for Disease Control and Prevention indicated that 1,716 HCWs were confirmed to be infected by SARS-CoV-2 in China.^6^ As of April 9, 2020, >5,000 HCWs have been infected in Italy^7^ and nearly 9,300 in the United States.^8^

To reduce the number of infections among HCWs and to achieve early identification and management, it is crucial to determine the epidemiological and clinical characteristics of these infections. However, studies on this issue are scarce thus far. Additionally, under the current context of heavy workload and continuing psychological stress placed on frontline HCWs,^9,10^ the critical question that needs to be addressed is whether there are differences in signs, symptoms, and case fatality rates between HCWs and other occupational groups. We conducted this study to address these questions.

## Methods

### Study design, data source, and study population

**Fig. 1.**
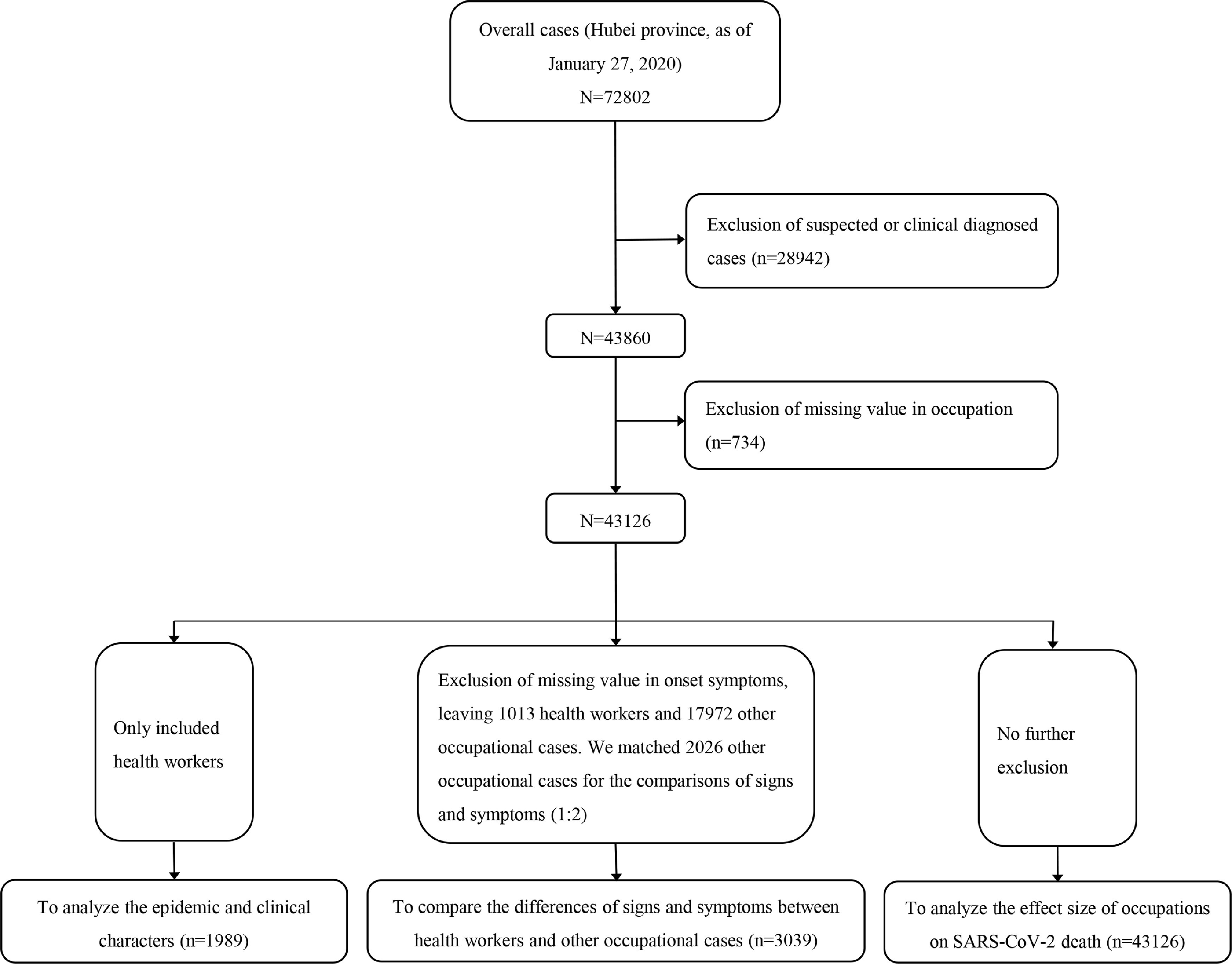
The flowchart of subjects.

The participants in this study were all cases identified through epidemiological investigation in Hubei Province as of February 27, 2020, who were followed until March 7, 2020. Individuals were classified as HCWs or “other” occupational cases based on their occupation.^6^ Specifically, HCW refers to medical and health professionals who are directly involved in patient care. These occupations include reception, screening, inspection, testing, transfer, treatment, nursing, epidemiological investigation and medical observation, as well as medical and health professionals who directly perform case specimen collection, pathogen detection, pathological examination, and pathological anatomy. The others are defined as other occupational cases. After excluding suspected or clinically diagnosed cases and those who had a missing value recorded for their occupation, 43,126 cases remained in the data set. First, we included HCWs only, and we analyzed the epidemiological and clinical characteristics of the subgroup of HCWs with COVID-19 (Fig. 1). Second, to compare the differences in signs and symptoms between HCWs and other occupational cases, we further excluded those with a missing value for the onset of symptoms variable. From the initial 43,126 cases, this left 1,013 HCWs and 17,972 other occupational cases. We then matched 2,026 other occupational cases based on sex, age, address and the date of symptom onset (Fig. 1). Third, for these 43,126 cases, and we analyzed the association between occupation and the risk of COVID-19 mortality (Fig. 1).

### Variables

Demographic characteristics and clinical features were collected using a case questionnaire at the time of diagnosis and were then input into the infectious disease information system by local epidemiologists or public health professionals. The case questionnaire contains basic demographic or epidemiological information (eg, sex, birthdate, present address, occupation, Wuhan-related exposure and confirmed cases exposure) and clinical information (eg, symptom onset, the date of symptom onset, blood cell count). We computed the age of each case using the date of symptom onset and birthdate. According to the present address, all of the records were further classed as residents in Wuhan or elsewhere. The severity of symptoms variable was categorized as asymptomatic, mild, common, severe, or critical. Our diagnostic criteria on clinical severity followed the Chinese clinical guidance for COVID-19 pneumonia diagnosis and treatment.^11^ All of the confirmed HCWs were diagnosed based on positive viral nucleic acid test results from throat swab samples.^6^ The date of symptom onset in asymptomatic cases was based on the date of the positive nucleic acid test results.

### Statistical analyses

Categorical variables were expressed as counts (alongside percentages), and continuous variables were expressed as medians (alongside interquartile ranges). All statistical analyses were performed using R version 3.5.3 software (R Foundation for Statistical Computing, Vienna, Austria). A 2-tailed *P* value <.05 was considered statistically significant.

To describe the epidemiological characteristics and clinical features of SARS-CoV-2 infection among HCWs, we classified the date of symptom onset into 4 categories: before January 23, 2020 (the lockdown date in Wuhan city), January 24–February 3, February 4–13, and February 14–27. Moreover, an epidemic curve for the distribution of case severity was constructed to show the progression of illness among HCWs involved in the outbreak over time.

To include controls in our study, we matched 1,013 HCWs and 2,026 other occupational cases based on sex, age, address, and the date of symptom onset, and we compared the differences in clinical signs or symptoms. The Wilcoxon signed-rank test was used to compare continuous variables with skewed distribution, and the χ^2^ test or the Fisher exact test was used to compare categorical variables.

A logistic regression model was performed to analyze the association between occupation and the risk of COVID-19 mortality. Odds ratios (ORs) and 95% confidence intervals (CIs) were calculated for the output. The potential confounders were ascertained based on prior publications^12,13^ and these included age range (20–29, 30–39, 40–49, 50–59, and 60+ years), sex (male or female), disease severity (severe or nonsevere), address (Wuhan city or elsewhere), and the date of symptom onset (before January 24–February 3, February 4–13, or February 14–27).

## Results

### Epidemiological and clinical characteristics of HCWs stratified by the date of symptom onset

From December 27, 2019, through February 27, 2020, a total of 1,989 HCWs were laboratory-confirmed to have SARS-CoV-2 infection in Hubei Province, China. The epidemiological and clinical characteristics of HCWs, stratified by the date of symptom onset, are shown in Table 1. Fever and cough were the most common onset symptoms in HCWs during any period. In total, 297 HCWs (14.93%) were diagnosed with severe or critical cases, and 18 HCWs had died by the end date of the follow-up period. Nearly half of HCWs had no reported contact with known COVID-19 patients (969, 48.72%). Before the date of lockdown in Wuhan city (January 23, 2020), a total of 587 HCWs were laboratory-confirmed to have SARS-CoV-2 infection, and most of these cases occurred in Wuhan city (445, 75.81%). From February 14 to February 27, a total of 142 HCWs were laboratory-confirmed to have SARS-CoV-2 infection, and asymptomatic cases accounted for 47.18% of the cases (68, 47.18%).

**Table 1.** Characteristics of 1,989 Healthcare Workers (HCWs)

Characteristic	Before 2020/01/23 (n=587)	01/24–02/03 (n=974)	02/04–02/13 (n=286)	02/14–02/27 (n=142)
Age, median (IQR)	38.08 (31.34–47.66)	38.76 (30.61–49.31)	37.18 (29.52–46.89)	33.82 (27.34–44.41)
20–29 y, no. (%)	111 (18.91)	217 (22.28)	79 (27.62)	57 (40.14)
30–39 y, no. (%)	218 (37.14)	302 (31.01)	84 (29.37)	38 (26.76)
40–49 y, no. (%)	136 (23.17)	223 (22.9)	76 (26.57)	23 (16.2)
50–59 y, no. (%)	90 (15.33)	154 (15.81)	26 (9.09)	19 (13.38)
≥60 y, no. (%)	32 (5.45)	78 (8.01)	21 (7.34)	5 (3.52)
Sex, male, no. (%)	230 (39.18)	324 (33.26)	74 (25.87)	39 (27.46)
Contact with confirmed patients, no. (%)	277 (47.19)	515 (52.87)	146 (51.05)	82 (57.75)
Clinical severity, no. (%)				
Asymptomatic	0 (0.00)	0 (0.00)	6 (2.10)	67 (47.18)
Mild	198 (33.73)	487 (50.00)	149 (52.10)	37 (26.06)
Common	264 (44.97)	342 (35.11)	109 (38.11)	33 (23.24)
Severe or critical	125 (21.29)	145 (14.89)	22 (7.69)	5 (3.52)
Wuhan city, no. (%)	445 (75.81)	635 (65.20)	190 (66.43)	68 (47.89)
**Signs and symptoms, no. (%)**				
Fever, °C	278 (76.16)	341 (71.04)	62 (65.26)	8 (10.96)
<37.5	20 (7.22)	34 (10.03)	6 (9.68)	2 (25)
37.5–37.9	84 (30.32)	143 (42.18)	38 (61.29)	5 (62.5)
38.0–38.4	69 (24.91)	78 (23.01)	6 (9.68)	0 (0)
38.5–38.9	71 (25.63)	61 (17.99)	9 (14.52)	0 (0)
≥39.0	33 (11.91)	23 (6.78)	3 (4.84)	1 (12.5)
Missing	310	635	224	134
Cough	194 (53.15)	258 (53.75)	47 (49.47)	13 (17.81)
Fatigue	156 (42.74)	137 (28.54)	16 (16.84)	4 (5.48)
Myalgia	88 (24.11)	98 (20.42)	6 (6.32)	2 (2.74)
Headache	61 (16.71)	77 (16.04)	7 (7.37)	1 (1.37)
Chest tightness	36 (9.86)	63 (13.12)	5 (5.26)	5 (6.85)
Sore throat	37 (10.14)	38 (7.92)	7 (7.37)	2 (2.74)
Chills	49 (13.42)	52 (10.83)	6 (6.32)	1 (1.37)
Diarrhea	34 (9.32)	30 (6.25)	7 (7.37)	1 (1.37)
Polypnea	35 (9.59)	27 (5.62)	1 (1.05)	1 (1.37)
Dyspnea	20 (5.48)	30 (6.25)	1 (1.05)	0 (0)
Arthralgia	28 (7.67)	27 (5.62)	0 (0)	0 (0)
Nasal obstruction	18 (4.93)	20 (4.17)	2 (2.11)	1 (1.37)
Nasal discharge	20 (5.48)	27 (5.62)	1 (1.05)	2 (2.74)
Vomit	13 (3.56)	15 (3.12)	2 (2.11)	1 (1.37)
White blood cell count <9.5, no. (%)	208 (94.98)	245 (98)	57 (96.61)	24 (100)
Lymphocytes,<1.0, no. (%)	93 (42.47)	102 (40.8)	19 (32.2)	1 (4.17)
Computed tomography, no. (%)	303 (85.35)	413 (85.51)	72 (75.79)	30 (41.1)
Death, no. (%)	6 (1.02)	12 (1.23)	0 (0)	0 (0)

**Fig. 2.**
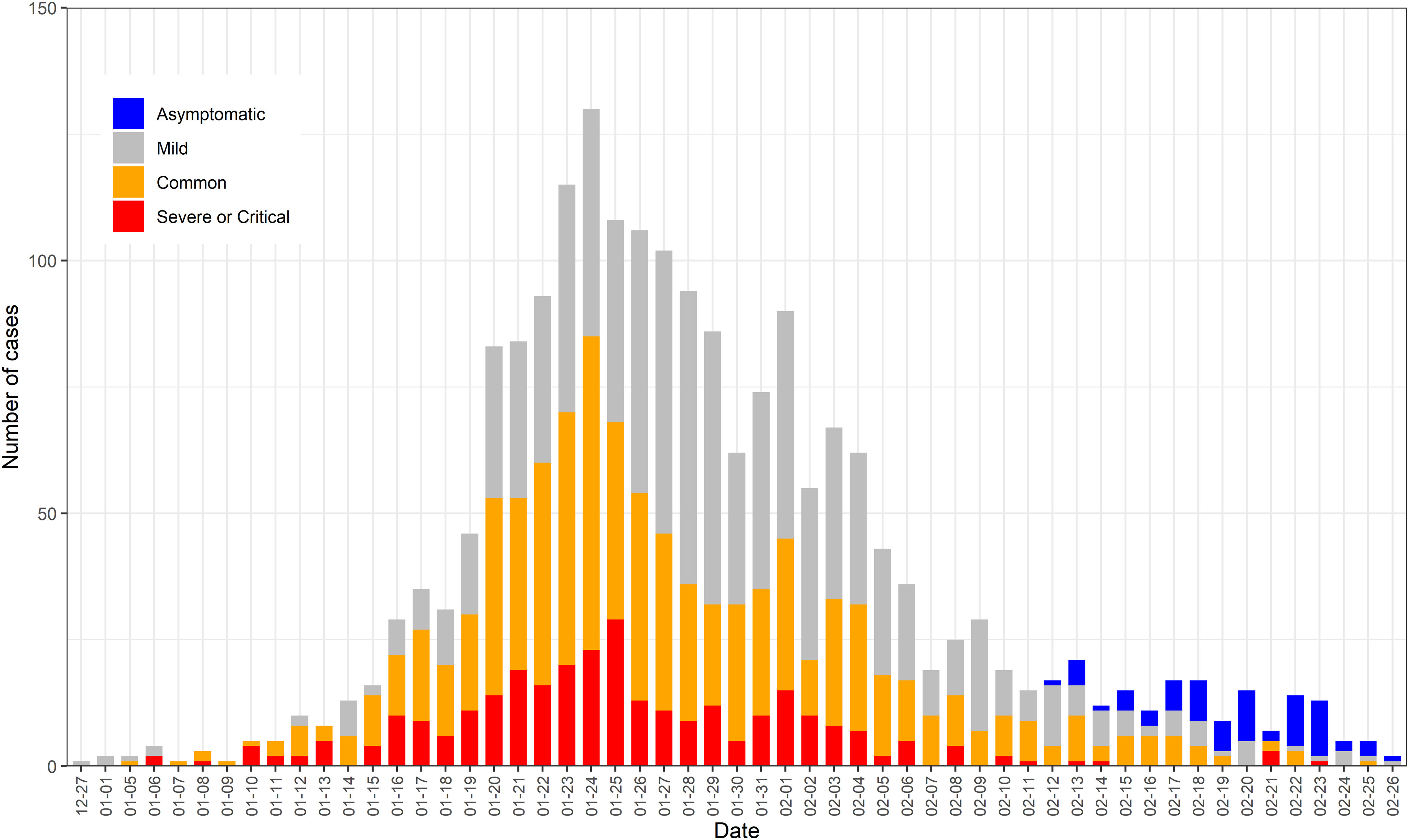
Epidemiological curve of SARS-CoV-2 in Hubei Province through February 27, 2020. Note. This epidemiological curve shows the progression of illness among HCWs in the outbreak over time.

We created an epidemiological curve to illustrate the progression of illness among HCWs involved in the outbreak over time (Fig. 2). Asymptomatic, mild, common, and severe or critical cases were stacked to show total daily cases by date of symptom onset. In general, the peak number of cases occurred around January 23–25, 2020. Thereafter, illness incidence declined.

### The comparison of clinical characteristics between HCWs and other occupational cases

Based on sex, age, address, and the date of symptom onset we matched 1,013 HCWs and 2,026 other occupational cases. The results of the comparison of clinical characteristics between the 2 groups are presented in Table 2. Fever and cough were the most prevalent symptoms at disease onset in both HCWs (689 [68.02%] and 512 [50.54%]) and other occupational cases (1,583 [78.13] and 1,095 [54.05%]). Compared with other occupational cases, HCWs had higher rates of fatigue (30.90% vs 25.02%; *P* < .001), myalgia (19.15% vs 13.43%; *P* < .001), and chills (10.66% vs 8.09%; *P* = .023), but they had a lower rate of dyspnea (5.03% vs 7.35%; *P* = .019). HCWs also had a (borderline) significantly lower case fatality rate (0.99% vs 2.02%; *P* = .052).

**Table 2.** The Comparison of Clinical Characteristics Between Healthcare Workers (HCWs) and the Matched Cases in Other Occupations

Characteristic	Other Occupational Cases (n=2,026)	HCWs (n=1,013)	*P* Value
Age, median y [IQR]	39.27 (30.48–49.23)	39.06 (30.84–48.89)	.764
20–29 y, no. (%)	465 (22.95)	221 (21.82)	
30–39 y, no. (%)	594 (29.32)	318 (31.39)	
40–49 y, no. (%)	511 (25.22)	246 (24.28)	
50–59 y, no. (%)	318 (15.7)	159 (15.7)	
≥60 y, no. (%)	138 (6.81)	69 (6.81)	
Sex, male, no. (%)	793 (39.14)	369 (36.43)	.158
Contact with confirmed patients, no. (%)	472 (23.3)	472 (46.59)	<.001
Severe or critical, no. (%)	287 (14.17)	151 (14.91)	.622
Wuhan city, no. (%)	734 (36.23)	367 (36.23)	1.000
**Signs and symptoms, no. (%)**			
Fever, °C	1,583 (78.13)	689 (68.02)	<.001
<37.5	121 (7.71)	62 (9.04)	.727
37.5–37.9	607 (38.66)	270 (39.36)	
38.0–38.4	364 (23.18)	153 (22.3)	
38.5–38.9	321 (20.45)	141 (20.55)	
≥39	157 (10)	60 (8.75)	
Missing	456	327	
Cough	1,095 (54.05)	512 (50.54)	.074
Fatigue	507 (25.02)	313 (30.9)	<.001
Myalgia	272 (13.43)	194 (19.15)	<.001
Headache	261 (12.88)	146 (14.41)	.267
Chest tightness	237 (11.7)	109 (10.76)	.480
Sore throat	145 (7.16)	84 (8.29)	.296
Chills	164 (8.09)	108 (10.66)	.023
Diarrhea	135 (6.66)	72 (7.11)	.703
Polypnea	161 (7.95)	64 (6.32)	.123
Dyspnea	149 (7.35)	51 (5.03)	.019
Arthralgia	78 (3.85)	55 (5.43)	.056
Nasal obstruction	95 (4.69)	41 (4.05)	.476
Nasal discharge	100 (4.94)	50 (4.94)	1.000
Vomit	65 (3.21)	31(3.06)	.912
White blood cell count <9.5, no. (%)	1,250 (95.2)	534 (96.74)	.173
Lymphocytes <1.0, no. (%)	454 (34.58)	215 (38.95)	.081
Computed tomography, no. (%)	1,661 (83.09)	812 (81.2)	.332
Death, no. (%)	41 (2.02)	10 (0.99)	.052

### The association between occupation (HCWs or other) and death with COVID-19

Of the overall laboratory-confirmed cases, those who were older, male, severely or critically ill, Wuhan residents, and cases with disease onset at early outbreak had significantly higher risk of death (Table S1). After adjustment for these confounders, HCWs had a lower risk of death than other occupational cases (OR, 0.50; 95% CI, 0.30–0.79) (Table 3).

**Table 3.** The Risk Factors of SARS-CoV-2 Deaths Among Laboratory-Confirmed Cases (n=43,126)

Characteristic	No. Deaths/ No. Survivors	OR (95% CI)^a^	*P* Value
**Age**			
20–29 y	11/3,963	Ref	…
30–39 y	31/6,497	1.32 (0.68–2.76)	.428
40–49 y	71/7,595	2.36 (1.30–4.72)	.008
50–59 y	258/9,486	6.13 (3.51–11.97)	<.001
≥60 y	1,703/13,511	22.3 (12.91–43.20)	<.001
**Sex**			
Male	1,323/20,385	Ref	…
Female	751/20,667	0.54 (0.49–0.60)	<.001
**Severe or critical**			
No	621/34,062	Ref	…
Yes	1,453/6,990	7.05 (6.37–7.80)	<.001
**Wuhan city**			
No	578/15,784	Ref	…
Yes	1,496/25,268	1.10 (0.99–1.22)	.084
**The date of symptom onset**			
Before 2020/01/23	664/7,999	Ref	…
01/24–02/03	1,074/20,724	0.76 (0.68–0.84)	<.001
02/04–02/13	278/8,319	0.52 (0.45–0.61)	<.001
02/14–02/27	58/4,010	0.30 (0.23–0.40)	<.001
**HCWs**			
No	2,056/39,081	Ref	…
Yes	18/1,971	0.50 (0.30–0.79)	.005

Note: OR, odds ratio; CI, confidence interval; HCWs, healthcare workers.

a Covariates in model included age, sex, Wuhan city, disease severity, occupations, and the date of symptom onset.

## Discussion

In the study cohort, 1,989 HCWs were infected by SARS-CoV-2, including 297 (14.93%) severe or critical cases and 73 (3.67%) asymptomatic cases. Fever and cough were the most prevalent symptoms at disease onset in both HCWs and other occupational cases. Compared with other occupational cases, HCWs had higher rates of fatigue (30.90% vs 25.02%; *P* < .001) and myalgia (19.15% vs 13.43%; *P* < .001). As of March 7, 2020, 18 HCWs and 2,056 other occupational cases died due to COVID-19, for case fatality rates of 0.9% and 5.0%, respectively. HCWs also had a significantly lower risk of death than other occupational cases (OR, 0.50; 95% CI, 0.30–0.79) after adjusting for potential confounders. To the best of our knowledge, this is the largest sample among the reports on the epidemiological and clinical characteristics of SARS-CoV-2 infections among HCWs in China.

Fever and cough were the dominant symptoms among HCWs at any period of time since the SARS-CoV-2 outbreak (Table 1), which is in line with recent findings based on the general population.^1,14,15^ Notably, HCWs had significantly higher rates of myalgia and fatigue than other occupational cases. One of the possible reasons for this may be that HCWs had heavier workloads, longer working hours, and higher work pressure than other occupations during this special period. Another plausible reason might be that HCWs have a more comprehensive understanding of the disease and may report more symptoms than other occupations.

Based on the data from the WHO, the case fatality rate of SARS-CoV-2 infection varies in different regions or countries. For instance, as of September 30, 2020, the case fatality rate was 11.5% in Italy, 9.4% in the United Kingdom, 8.7% in Belgium, 6.4% in Sweden, 5.8% in France, 5.6% in Netherlands, 4.2% in Spain, and 2.9% in America.^16^ In China, the official data presented a case fatality rate of 5.2% (4,746 deaths in 91,041 confirmed cases). However, data to show the case fatality rate of SARS-CoV-2 among HCWs are limited, and no study has yet indicated whether HCWs have a higher risk of death from COVID-19. Our study results contributed to the current literature, showing that through March 7, 2020, the case fatality rate of 1,989 infected HCWs was 0.9% in Hubei Province of China and that HCWs had a significantly lower risk of death from COVID-19. Generally, frontline HCWs experienced heavier workloads and higher psychological stress than other occupational cases during this pandemic period. HCWs have a more comprehensive understanding of diseases and are, on average, younger than those in other occupations, and potentially important protective factors may be related to these factors. Another potential explanation might be the easy access to medical resources that comes with being a HCW.

Notably, nearly half of HCWs with confirmed COVID-19 reported no known contact with COVID-19 patients, implying that the potential infection risks do not only come from the hospital environment (eg, patients, other HCWs, or fomites), but also from community transmission. These findings are in line with recent findings in Singapore.^17^ According to previous studies,^18,19^ nosocomial infection of SARS in HCWs was affected by clinical condition of SARS patients, hospital environments, and their personal protective measures. Therefore, training for HCWs, especially in densely populated areas or countries with insufficient medical resources and insufficient experience in prevention or treatment of infectious diseases, should be conducted.

Moreover, our findings suggest that although only a small proportion of HCWs are asymptomatic infections, most of them occurred in the late stage of the outbreak. The explanation might be, at least in part, the result of testing protocols that were used to identify symptomatic cases in the early stage of the outbreak.^20^ Additionally, with the improvement of medical resources, HCWs were more likely to seek testing at an earlier stage of illness or to seek testing while asymptomatic and after exposure to confirmed or suspected COVID-19 cases. Non–symptom-based screening for HCWs also indicated that most infected HCWs were asymptomatic.^21^ Given the transmission possibility of asymptomatic cases,^22^ achieving early detection and expanding the scope of COVID-19 testing among HCWs might help to improve early isolation and early management. Early detection contributes to a reduction in infection rate. Therefore, local public health departments and hospitals should strengthen their detection strategies among HCWs to reduce the risk of nosocomial infection, to minimize clusters among HCWs, and to avoid transmission to patients.

This study has several limitations. First, since HCWs are more likely to be tested earlier in the course of disease than other occupational groups, the potential for detection bias and changes in testing protocols over the course of the study period may have influenced our findings. Second, although some clinical laboratory indicators (eg, blood gas and inflammatory markers) have been reported to be associated with an increased risk of mortality,^23^ the absence of these data might bias the estimated association in the present study. Third, the incubation period could not be estimated here. Lastly, the existence of recall bias (eg, the date of symptoms onset) might inevitably affect our assessment.

In summary, compared with cases in other occupation groups, our results suggest that HCWs have a lower risk of death in the Hubei Province but have higher rates of fatigue and myalgia. Nearly half of HCWs with confirmed COVID-19 reported no known contact with COVID-19 patients, which highlights the need to maintain strict vigilance and precautionary measures at all times. Additionally, to protect the health and safety of this essential national workforce, it is crucial to strengthen the detection strategies and to reinforce infection control among HCWs.

## Supplementary material

For supplementary material accompanying this paper visit http://dx.doi.org/10.1017/ice.2020.1321. click here to view supplementary material
