# Supplementary material for: Epidemiological and clinical characteristics of severe acute respiratory coronavirus virus 2 (SARS-CoV-2) infection among healthcare workers in Hubei Province, China
Source: Infect Control Hosp Epidemiol. 2020 Nov 18:1–7. doi: 10.1017/ice.2020.1321 (PMC7691647; doi:10.1017/ice.2020.1321)
Supplement: Supplementary file 1 [file S0899823X20013215sup001.docx]

**Epidemiological and clinical characteristics of SARS-CoV-2 infection among healthcare workers in Hubei Province of China**

Mingyang Wu, PhD ^1, †^, Cong Xie, PhD ^2, †^, Ran Wu, PhD ^2, †^, Yanling Shu, MD ^3, †^, Lulin Wang, PhD ^1^, Mingyan Li, PhD ^2, *^, Youjie Wang, PhD ^1^

**Table S1 The crude fatality rate of SARS-CoV-2 infections among subgroup population (n=43,126).**

|  | Survivors (n=41,052) | Deaths (n=2074) | Case fatality rate (%) | *P* |
| --- | --- | --- | --- | --- |
| Age, year | 52.35(39.66-63.64) | 70.67(62.95-78.62) | -- | <0.001 |
| 20-29 | 3963 | 11 | 0.28 |  |
| 30-39 | 6497 | 31 | 0.47 |  |
| 40-49 | 7595 | 71 | 0.93 |  |
| 50-59 | 9486 | 258 | 2.65 |  |
| 60+ | 13511 | 1703 | 11.19 |  |
| Sex, No. |  |  |  | <0.001 |
| male | 20385 | 1323 | 6.09 |  |
| female | 20667 | 751 | 3.51 |  |
| Severe or Critical, No. |  |  |  | <0.001 |
| no | 34062 | 621 | 1.79 |  |
| yes | 6990 | 1453 | 17.21 |  |
| Wuhan city, No. (%) |  |  |  | <0.001 |
| no | 15784 | 578 | 3.53 |  |
| yes | 25268 | 1496 | 5.59 |  |
| The date of symptom onset, No. | |  |  | <0.001 |
| before 2020/01/23 | 7999 | 664 | 7.66 |  |
| 01/24-02/03 | 20724 | 1074 | 4.93 |  |
| 02/04-02/13 | 8319 | 278 | 3.23 |  |
| 02/14-02/27 | 4010 | 58 | 1.43 |  |
| Healthcare workers, No. |  |  |  | <0.001 |
| no | 39081 | 2056 | 5.0 |  |
| yes | 1971 | 18 | 0.9 |  |
